# Facilitators and barriers to chlamydia testing in general practice for young people using a theoretical model (COM-B): a systematic review protocol

**DOI:** 10.1136/bmjopen-2016-013588

**Published:** 2017-03-09

**Authors:** Lorraine K McDonagh, John M Saunders, Jackie Cassell, Hamad Bastaki, Thomas Hartney, Greta Rait

**Affiliations:** 1 National Institute for Health Research Health Protection Research Unit in Blood Borne and Sexually Transmitted Infections at University College London, London, UK; 2 Research Department of Primary Care and Population Health, University College London, London, UK; 3 National Chlamydia Screening Programme, Public Health England, London, UK; 4 Division of Primary Care and Public Health, Brighton and Sussex Medical School, University of Brighton, Brighton, UK

**Keywords:** chlamydia, general practice, young people, behavior change, PRIMARY CARE

## Abstract

**Introduction:**

Chlamydia is a key health concern with high economic and social costs. There were over 200 000 chlamydia diagnoses made in England in 2015. The burden of chlamydia is greatest among young people where the highest prevalence rates are found. Annual testing for sexually active young people is recommended; however, many of those at risk do not receive testing. General practice has been identified as an ideal setting for testing, yet efforts to increase testing in this setting have not been effective. One theoretical model which may provide insight into the underpinnings of chlamydia testing is the Capability, Opportunity and Motivation Model of Behaviour (COM-B model). The aim of this systematic review is to: (1) identify barriers and facilitators to chlamydia testing for young people in general practice and (2) use a theoretical model to conduct a behavioural analysis of chlamydia testing behaviour.

**Methods and analysis:**

Qualitative, quantitative and mixed methods studies published after 2000 will be included. Seven databases (MEDLINE, PubMed, EMBASE, Informit, PsycInfo, Scopus, Web of Science) will be searched to identify peer-reviewed publications which examined barriers and facilitators to chlamydia testing in general practice. Risk of bias will be assessed using the Critical Appraisal Skills Programme. Data regarding study design and key findings will be extracted. The data will be analysed using thematic analysis and the resultant factors will be mapped onto the COM-B model components. All findings will be reported in accordance with the Preferred Reporting Items for Systematic Reviews and Meta-Analyses guidelines.

**Ethics and dissemination:**

Ethical approval is not required. The results will be disseminated via submission for publication to a peer-review journal when complete and for presentation at national and international conferences. The review findings will be used to inform the development of interventions to facilitate effective and efficient chlamydia testing in general practice.

## Introduction

Chlamydia is a key public health concern with great economic and social costs. There were 200 288 chlamydia diagnoses made in England in 2015.^1^ The burden of chlamydia is greatest among people aged 15–24 years where the highest prevalence rates are found.^1^ Chlamydia is often asymptomatic and can pose severe health consequences if left undiagnosed and/or untreated (ie, pelvic inflammatory disease, infertility, ectopic pregnancy). Testing and early treatment, therefore, are an effective way to interrupt the transmission chain in the population and prevent such sequelae.^2^

### Chlamydia testing in general practice

In 2015 (last complete year of sexually transmitted infection surveillance data in England), a total of 1 538 820 chlamydia tests were conducted in 15 to 24-year olds; 298 263 (19.4%) of these were performed in general practice.^1^ The test positivity (number of positive tests divided by total number of tests) in general practice is slightly lower than the average for all tests in young people, ∼5.9% versus 8.3%, respectively.^1^ This indicates that testing in general practice reaches a slightly different risk group compared to specialist settings. Additionally, many more young people attend general practice compared to sexual health clinics over the course of a year. Hence, there is considerable potential to reach more young people with testing in general practice compared to other settings. In the UK, STI testing is funded by local authorities (local government) and there is currently a drive to shift high volume, low cost testing (ie, chlamydia testing in asymptomatic young people) away from expensive specialist settings and into primary care (eg, general practice).^3^ This would free up capacity in specialist settings to see more complex patients and put the onus on general practice to do more testing in asymptomatic young people.

General practice is one logical setting for chlamydia testing for a variety of reasons. Over 60% of young people attend general practice annually.^4^ ^5^ Young people have reported a preference to receive testing and testing results from a general practitioner.^6–9^ Higher rates of positivity have been found, particularly for men, in general practice compared to non-healthcare settings such as universities.^3^ ^10^ Finally, regular screening is easier to maintain in this setting, due to patients attending for other reasons, which is necessary for continued transmission reduction.^10^

### Barriers and facilitators to chlamydia testing

Annual testing for sexually active young people is recommended in several countries including Australia, Denmark, England, Norway, Sweden and the USA.^11–15^ Unfortunately, however, many of those at risk do not receive testing. Lack of testing has been attributed to barriers at the patient level, provider level and system level. In a recent narrative review of chlamydia testing in general practice, the most common barriers identified were the social context of testing (ie, stigma), poor knowledge/training and time constraints.^16^ However, the review was conducted using a narrative approach, and thus lacks the rigorous methodological techniques of the systematic review. It is possible that potentially relevant studies were missed.

To overcome the barriers to testing and exploring the facilitators, numerous interventions using a variety of strategies have been conducted.^17^ ^18^ The evidence for their effectiveness is mixed. For those that have been reported as being effective, the effects tend to be modest,^19^ ^20^ or demonstrate little clinical significance.^21^ One possible explanation for these disappointing results is the lack of input from theories of behaviour.

### The role of theory

It is increasingly recognised that an understanding of behaviour and behaviour change is required to maximise the effectiveness of interventions.^22^ ^23^ Essentially, in order to change a particular behaviour (such as increase chlamydia testing), it is necessary to have a theoretical understanding of that behaviour.^24^ ^25^ Applying theory to intervention design allows researchers to explain and predict specific behaviours in terms of why, when and how they occur, as well as which factors should be targeted in order to alter them. There are numerous theories of behaviour and it is unclear which one to choose. A further issue is that, once a suitable theory is identified, it can be difficult to decipher how to apply it to the development of an intervention.^26^

**Figure 1:**
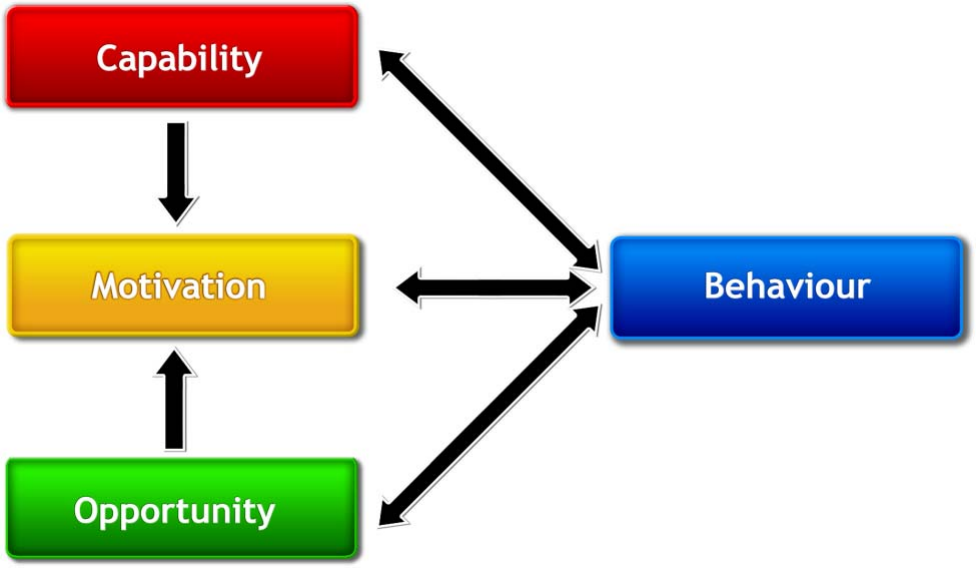
The COM-B Model.^26^

One promising overarching theory of behaviour, and basis for designing interventions aimed at behaviour change, is the Capability, Opportunity, Motivation, Behaviour (COM-B model).^27^ The COM-B model proposes that behaviour (B) is the result of an interaction between three components: capability (C), opportunity (O) and motivation (M). Behaviour change, therefore, requires a change in one or more of these components. The COM-B model lies at the centre of the Behaviour Change Wheel (a tool kit for designing behaviour change interventions,^27^) and is the starting point of intervention development. Capability can be psychological (eg, knowledge) or physical (eg, skills); opportunity can be social (eg, societal influences) or physical (eg, environmental resources); motivation can be automatic (eg, emotion) or reflective (eg, underlying beliefs, intentions^25–27^). In other words, for a person to engage in a specific behaviour, they need to: (1) be psychologically and physically able to do the behaviour; (2) have the physical and social opportunity to do the behaviour; and (3) want or need to do the behaviour. The model is illustrated in figure 1.

The COM-B model has not yet been applied to chlamydia testing; however, it has been successfully applied in other health behaviour contexts,^28–35^ and has been used as basis for developing effective interventions.^36–39^ The benefit of employing the COM-B model to chlamydia testing is that several distinct explanatory components are outlined; thus, additional potential influences on behaviour can be considered which is essential for the development of an intervention. Furthermore, once the COM-B model has been used to conduct an in-depth theoretically-based analysis of the behaviour in question, it can be ultimately used to identify the mediators and moderators of behaviour to be targeted by an intervention with the Behaviour Change Wheel.^26^

### Research aims

The aim of this systematic review is to identify the barriers and facilitators to chlamydia testing for young people in general practice and to use the COM-B model to conduct a behavioural analysis of chlamydia testing. The specific research questions of this systematic review are:

1. What are the facilitators and barriers to chlamydia testing for young people in general practice?
2. What are the facilitators and barriers to chlamydia testing for primary care providers in general practice?
3. How do identified facilitators and barriers of chlamydia testing for young people in general practice map on to a theoretical model of behaviour change?
4. How do identified facilitators and barriers of chlamydia testing for primary care providers in general practice map on to a theoretical model of behaviour change?

## Methods and analysis

This systematic review will be conducted in accordance with the Preferred Reporting Items for Systematic Reviews and Meta-Analyses (PRISMA) Statement guidelines.^40^ The PRISMA-Protocol checklist is presented in online supplementary appendix 1. In addition, the relevant literature for reporting of qualitative studies within systematic reviews will be consulted to ensure that all necessary information is provided.^41^ ^42^ This review is registered with the international database of prospectively registered systematic reviews in health and social care (PROSPERO; registration number CRD42016041786; available at http://www.crd.york.ac.uk/PROSPERO/display_record.asp?ID=CRD42016041786).

10.1136/gutjnl-2015-311146.supp1 Supplementary appendix

### Eligibility criteria

To be included in the review, papers will have to meet the following Population, Intervention, Context, Outcomes and Study design elements:

#### Population

Inclusion criteria:

- Young men and women (aged 15–24 years) and primary care providers (general practitioners, practice nurses, nurse practitioners).

Exclusion criteria:

- Studies focusing exclusively on commercial sex workers, incarcerated people, people living with HIV, victims of sexual or domestic abuse or violence, intravenous drug users and individuals with no fixed address as these groups have distinct needs beyond the scope of the review. Studies which partially include these populations (ie, as part of a general population sample) will be included; however, the sample composition will be discussed when interpreting their findings.

#### Intervention

The issue to be reviewed is opportunistic and systematic chlamydia testing for young people in general practice. Opportunistic testing will be defined as the offer of a diagnostic test to people attending general practice during a consultation for another reason. Systematic testing will be defined by the use of existing population registers to invite the target group to submit self-collected samples by post. A barrier will be defined as a factor that obstructs or prevents chlamydia testing; a facilitator will be defined as a factor that supports or promotes chlamydia testing.

Inclusion criteria:

- Randomised and non-randomised controlled trials, pretest and post-test designs, non-experiment observational (cross-sectional, case-series, case studies) and qualitative papers (interviews, focus groups).

Exclusion criteria:

- Exclusively set outside of general practice, exclusively focused on partner notification, campaigns exclusively focused on health promotion, and testing for diagnostic purposes when symptoms are present.

#### Context

Inclusion criteria:

- Studies conducted in Australia, Denmark, Ireland, the Netherlands, New Zealand and the UK as the model of delivering healthcare in general practice is comparable.

Exclusion criteria:

- Studies conducted in countries where the general practice setting is not comparable to that of the UK (eg, the USA, Canada).

#### Outcomes

Primary outcomes

- Young people: Perceived facilitators to chlamydia testing, perceived barriers to chlamydia testing, views towards chlamydia testing and acceptability of chlamydia testing.
- Primary care providers: Perceived facilitators to chlamydia testing, perceived barriers to chlamydia testing, views towards chlamydia testing and acceptability of chlamydia testing.

Secondary outcomes

- Classification of the identified barriers and facilitators into the subcomponents of the COM-B model: psychological capability, physical capability, social opportunity, physical opportunity, automatic motivation and reflective motivation..

#### Study design

Inclusion criteria:

- Quantitative (ie, cross-sectional, case-series and case studies), qualitative and mixed method studies.

Exclusion criteria:

Commentary or opinion publications that do not present new data.

### Information sources

The review will access both published and unpublished material by searching literature sources listed below between January 2000 and March 2016. Pre-2000 studies will be excluded as Nucleic Acid Amplification Tests of urine samples were introduced around this time, thus widening testing to non-clinical settings. The following databases will be searched: MEDLINE, PubMed, EMBASE, Informit, Web of Science, PsycINFO and Scopus. Relevant articles will also be identified from a manual search of reference lists of included articles.

### Search strategy

Medical Subject Headings (MeSH), subject headings and keywords will be created by using language that describes facilitators and barriers to chlamydia testing in general practice. Boolean combinations will create more specific searches. Initial scoping searches will be conducted to refine the search strategy. For example, key publications in the field will be identified and searches run to ensure that these are captured. The three sets of search terms relate to the context (general practice), the intervention (chlamydia testing) and outcomes (barriers and facilitators). The search strategy presented in online supplementary appendix 2 will be used to search MEDLINE, using an Ovid platform. Search terms pertaining to behaviour and behaviour change theories which will be piloted are presented in online supplementary appendix 3. Search terms will be modified for other databases where subject heading indexing differs from the terms used in MEDLINE.

10.1136/gutjnl-2015-311146.supp2 Supplementary appendix

10.1136/gutjnl-2015-311146.supp3 Supplementary appendix

### Data extraction and management

Data will be extracted from all full-text studies that fulfil the inclusion criteria. The reviewers will characterise the research design used in each study, including study population, sample size, response rate (if described), randomisation (if randomised controlled trial), presence or absence of a comparison group, data collection methods and key findings (primary/secondary outcomes).

A standardised framework will be devised and used to record the aims, methodological characteristics, main findings and relevance of each study. All identified references will be stored in Endnote. Data extraction will be undertaken by one reviewer (LMD) and checked by a second reviewer (HB/TH). Any discrepancies will be resolved by discussion between two researchers or adjudication by a third reviewer (GR/JC) when necessary. If required, primary authors will be contacted for additional data.

All studies that meet the inclusion criteria will be described in terms of:

- Design and quality, data collection methods, modes and techniques; validity of tools; qualitative, statistical and other analyses
- Participants, demographic characteristics (eg, age, ethnicity)
- Setting and recruitment methods, details of modes of delivery and any other aspects of content
- Theoretical framework employed in study (if any)

The following data will be extracted:

1. Data relating to young people:
  ▸ Perceived facilitators to chlamydia testing, perceived barriers to chlamydia testing, reasons for accepting or refusing the offer of chlamydia testing and acceptability of chlamydia testing in general practice.
  2. Data relating to primary care providers:
  ▸ Perceived facilitators to chlamydia testing, perceived barriers to chlamydia testing, provider reasons for providing chlamydia testing to young people and acceptability of chlamydia testing in general practice.

### Risk of bias (quality) assessment

The quality of each paper will be assessed independently by two reviewers (LMD and HB/TH). Any discrepancies will be resolved by consensus and, if necessary, a third party will be consulted. Each paper will be assessed using criteria based on the Critical Appraisal Skills Programme.^43^ Individual studies will be classified as primary (high-quality studies providing theoretical insight into sexual behaviour or thorough descriptions of particular contexts) and secondary (lower quality studies that had simple, non-detailed descriptions or do not support statements with evidence). The critical appraisal process will not be used to exclude papers prior to the synthesis; rather, it will be used to provide a context for the interpretation of the synthesised findings.

### Data synthesis and analysis

Individual study characteristics and outcomes will be summarised and presented in an evidence table. Thematic analysis, employing expert guidelines,^44^ will be used to identify prominent/recurrent themes in the literature. The use of the statistical software package NVivoV.11 will aid in managing the coding of the data set, with each code (or node) representing the emergent themes, for example, ‘education’. The frequency of themes as well as their explanatory value will be assessed. The themes will be refined through discussion and the use of constant comparison within and between codes to ensure that they accurately reflect the material.

Finally, a behavioural analysis of chlamydia testing behaviour will be conducted. Specifically, the identified themes will be classified into the six subcomponents of the COM-B model (psychological capability, physical capability, social opportunity, physical opportunity, automatic motivation and reflective motivation; see figure 1). Data classification will be conducted by one reviewer (LMD) in consultation with members of the review team (JS, JC, HB, TH and GR), employing guidelines set out by Michie and colleagues.^27^ Any discrepancies will be resolved by consensus.

## Discussion and dissemination

To the best of the authors' knowledge, this is the first systematic review to conduct a theoretical behavioural analysis of barriers and facilitators to chlamydia testing for young people in general practice. A theoretically based framework will be generated which will provide a greater insight into the complexities of chlamydia testing. The findings will have relevance to healthcare professionals, policymakers and commissioners in informing how best to improve the sexual health of young people. Importantly, the results will be integral to inform the development of interventions that will facilitate effective and efficient access to care and treatment for chlamydia in primary care, with the aim of reducing morbidity and transmission of chlamydia.

The review results will be disseminated via submission for publication to a peer-review journal when complete and submissions to be presented at national and international conferences (where eligible). Furthermore, lay and scientific summaries will be produced for wider dissemination (eg, via newsletters, blogs and organisation meetings).
