# Supplementary material for: Facilitators and barriers to chlamydia testing in general practice for young people using a theoretical model (COM-B): a systematic review protocol
Source: BMJ Open. 2017 Mar 9;7(3):e013588. doi: 10.1136/bmjopen-2016-013588 (PMC5353274; doi:10.1136/bmjopen-2016-013588)
Supplement: Supplementary appendix [file bmjopen-2016-013588supp_appendix2.pdf]

Appendix 2: Search Strategy for MEDLINE (via Ovid platform)

1. chlamydia*.tw.
2. c trachomatis.tw.
3. exp Chlamydia Infections/
4. exp Chlamydia trachomatis/
5. 1 or 2 or 3 or 4
6. screen*.tw.
7. detect*.tw.
8. test.tw.
9. tests.tw.
10. testing.tw.
11. diagnos*.tw.
12. 6 or 7 or 8 or 9 or 10 or 11
13. general practice* .tw.
14. general practitioner*.tw.
15. GP.tw.
16. primary care.tw.
17. family practice.tw.
18. family practitioner*.tw.
19. family medicine.tw.
20. family physician.tw.
21. primary health care.tw.
22. primary healthcare.tw.
23. primary care nurs*.tw.
24. general practice nurs*.tw.

25. nurse practictioner*.tw.
26. 13 or 14 or 15 or 16 or 17 or 18 or 19 or 20 or 21 or 22 or 23 or 24 or 25
27. barrier*.tw.
28. enabler*.tw.
29. facilitator*.tw.
30. attitude*.tw.
31. feasibility.tw.
32. 27 or 28 or 29 or 30 or 31
33. 5 and 12 and 26 and 32
34. limit 33 to yr="2000 – 2016"
