# Supplementary material for: Facilitators and barriers to chlamydia testing in general practice for young people using a theoretical model (COM-B): a systematic review protocol
Source: BMJ Open. 2017 Mar 9;7(3):e013588. doi: 10.1136/bmjopen-2016-013588 (PMC5353274; doi:10.1136/bmjopen-2016-013588)
Supplement: Supplementary appendix [file bmjopen-2016-013588supp_appendix3.pdf]

Appendix 3: Search terms for behaviour and behaviour change theories

1. acculturation theory
2. AIDS risk reduction model
3. behavior change
4. behavior change model
5. behavior change theories
6. behavior change theory
7. behavior change wheel
8. behavior economic theories
9. behavior modification
10. behavior theories
11. behavioral intervention
12. behavioral interventions
13. behaviour change
14. behaviour change model
15. behaviour change theories
16. behaviour change theory
17. behaviour change wheel
18. behaviour economic theories
19. behaviour modification
20. behaviour theories
21. behavioural intervention
22. behavioural interventions
23. capability
24. communication theory

25. community organisation theory

26. community organization theory

27. consumer information processing model

28. control theory

29. critical consciousness

30. cultural change

31. cultural changes

32. decisional balance theory

33. ecological model

34. ecological perspective

35. empowerment theory

36. enculturation theory

37. exchange theory

38. fear arousal theory

39. goal setting theory

40. goal theory

41. group level effect

42. group level effects

43. habit theory

44. health behavior theory

45. health behaviour theory

46. health belief model

47. health promotion theories

48. health promotion theory

49. innovation-decision process

50. interactionist model
51. intrapersonal theory
52. intrinsic motivation theories
53. mediation effects on behavior
54. mediation effects on behaviour
55. motivation
56. multicomponent stage model
57. natural recovery
58. normative change
59. normative changes
60. operant learning theory
61. operant theory
62. opportunity
63. organisational change theory
64. organizational change theory
65. personality theory
66. precaution adoption process
67. protection motivation theory
68. reasoned action approach
69. reasoned-action approach
70. reciprocal causality
71. reciprocal determinism
72. risk behavior theory
73. risk behaviour theory
74. self regulation theory

75. self determination theory
76. self-efficacy theory
77. self-perception theory
78. self-regulation theory
79. social capital
80. social change
81. social changes
82. social cognition model
83. social cognitive theory
84. social comparison theory
85. social determinism
86. social development
87. social developments
88. social influence
89. social learning theories
90. social learning theory
91. social marketing theory
92. social structural theory
93. social support
94. stage model
95. stage of change model
96. stages of change model
97. systems theory
98. theories of planned behaviour
99. theories of planned behaviour

100. theory of planned behaviour
101. theory of planned behaviour
102. theory of reasoned action
103. transtheoretical model
104. value-expectancy theory
